# Myocardial Microvascular Physiology in Acute and Chronic Coronary Syndromes, Aortic Stenosis, and Heart Failure

**DOI:** 10.1155/2022/9846391

**Published:** 2022-01-26

**Authors:** Alf I. Larsen, William F. Fearon, Todd J. Anderson, Nico Pijls

**Affiliations:** ^1^Department of Cardiology, Stavanger University Hospital, University of Bergen, Stavanger, Norway; ^2^Stanford University, Stanford, CA, USA; ^3^Libin Cardiovascular Institute, Cumming School of Medicine, University of Calgary, Calgary, Canada; ^4^Catharina Hospital, Eindhoven, Netherlands

Myocardial ischemia occurs when myocardial oxygen demand exceeds the coronary blood supply. The etiology is usually atherosclerotic obstructive epicardial coronary artery disease (CAD) presenting with the features of chronic coronary syndrome (CCS). Fractional flow reserve (FFR) has become the gold standard for assessing myocardial ischemia due to coronary artery stenosis. As demonstrated in the DEFER study, long-term prognosis after deferral of PCI of an intermediate coronary stenosis based on FFR >/ = 0.75 is excellent. The risk of cardiac death or myocardial infarction related to this stenosis is <1% per year and not decreased by stenting [1]. In the FAME study, it was shown that routine guidance of revascularization with measurement of FFR in patients with multivessel coronary artery disease, who are undergoing PCI with drug-eluting stents, significantly reduced the rate of the composite endpoint of death, nonfatal myocardial infarction, and repeat revascularization at 1 year [2]. In the FAME 2 trial, the investigators extended their findings by showing that in patients with stable coronary artery disease, FFR-guided PCI, as compared with medical therapy alone, improved the outcome. Furthermore, patients without ischemia had a favorable outcome with medical therapy alone [3].

These studies are dependent on the use of full hyperemia using vasodilating agents like adenosine. As a non-hyperemic surrogate to FFR, the so-called instantaneous wave-free ratio (iFR) and a number of comparable non-hyperemic pressure indices have been proposed [4]. Although these indices have been demonstrated to be noninferior to FFR in studies in relatively low-risk patients (Define Flair [5] and Swede Heart [6]), these are not as well validated and lack the clinical outcome data existing for FFR [7]. In the current issue of the journal, Ebihara et al. explored the effect of rate pressure product (RPP) on instantaneous wave-free ratio (iFR) [8]. By adding these extra parameters, the values might be more accurate and reproducible. They found that the best cutoff value of the iFR for predicting an FFR of 0.8 was 0.90 for all lesions. However, when the study population was divided into the low-RPP and high-RPP groups according to the median RPP, they found different iFR values predicting an FFR of 0.8, 0.93 for the low-RPP group and 0.82 for the high-RPP group. Consequently, the RPP has been demonstrated to affect the relationship between the FFR and iFR. With FFR as the gold standard, the iFR may underestimate and overestimate the functionality of ischemia in the low- and high-RPP groups, respectively.

However, with this being said, the angiographic evidence of “normal” or mildly diseased epicardial coronary arteries, usually defined as the absence of a luminal diameter reduction of <50% (or <70% of the luminal area reduction), is a common finding. This condition is usually defined as ischemia with no obstructive coronary artery (INOCA) disease and is likely related to the so-called coronary microcirculatory or microvascular dysfunction (CMD). Angina with no obstructive coronary arteries (ANOCAs) is the clinical term when a clinical diagnosis of ischemia is made in a patient without significant obstructive coronary artery disease, without the necessity of having demonstrated inducible ischemia. In reported studies on ANOCA, ischemia has been demonstrated in approximately 50% of the patients. Nevertheless, the two terms INOCA and ANOCA are often interchangeably used.

## 1. Epidemiology

Up to 40% of patients undergoing coronary angiography with signs and symptoms of angina pectoris are characterized with INOCA [9]. In the American College of Cardiology National Cardiovascular Data Registry from January 2004 through April 2008, at 663 hospitals, slightly more than one-third of patients without known disease who underwent elective cardiac catheterization had obstructive coronary artery disease. The authors of this report suggested better strategies for risk stratification to inform decisions and to increase the diagnostic yield of cardiac catheterization in routine clinical practice [7]. Moreover, estimates from the WISE database indicate that there are at least 3-4 million patients in the USA with signs and symptoms of ischemia despite no evidence of obstructive CAD [10]. However, this might be an old fashion approach to the problem of recurrent angina. In patients with residual angina or recurrence of angina after percutaneous coronary intervention (PCI), functional mechanisms are responsible for the vast majority of cases [11]. The large proportion of patients with angina and near-normal or normal coronary angiogram is thus a large challenge for the cardiology society because a vast number of patients are not appropriately diagnosed. Recently, a large study using intracoronary flow wires in 151 patients with INOCA demonstrated microvascular dysfunction in approximately 75% of the patients [12].

## 2. Definition

In 1988, Cannon and Epstein proposed that dysfunction of small intramural prearteriolar coronary arteries might be the pathogenic cause of a syndrome introduced as “microvascular angina” (MVA) in this patient population. This condition was characterized by heightened sensitivity of the coronary microcirculation to vasoconstrictor stimuli and a limited microvascular vasodilator capacity [13], mainly caused by dysfunction of small intramural prearteriolar coronary arteries [14]. Tests to identify this syndrome are typically performed using mediators of full hyperemia—adenosine or dipyridamole. However, functional etiology for angina also comprises endothelial dysfunction-associated vasospasm of large epicardial arteries. In 1959, Prinzmetal and his colleagues described a syndrome characterized by angina at rest, with transient ST-segment elevation, in patients with diseased coronary arteries [15].

This might be diagnosed during provocation tests with acetylcholine during coronary angiography. Acetylcholine binds vascular muscarinic acetylcholine receptors inducing endothelial NO release with subsequent arterial dilatation when endothelial function is intact. However, in the presence of endothelial dysfunction, acetylcholine induces conduit vessel arterial constriction due to direct smooth muscle cell constriction.

Reproduction of typical symptoms, ECG changes, and angiographically verified vasospasm is diagnostic [16]. Unlike the focal spasm and ST elevation seen with classic Prinzmetal's angina, diffuse vasospasm is the usual pattern with endothelial dysfunction detected with acetylcholine. In the CorMica study, a vasospastic pattern was seen in about ¼ of the INOCA cohort [17]. Many subjects will have both microvascular and conduit vessel abnormalities. Finally, typical symptoms and ECG alterations might also occur without obvious changes of the coronary angiogram in response to acetylcholine indicating small-vessel vasospasm. All of these conditions are more or less associated with the progressive process of coronary atherosclerosis initiated by endothelial dysfunction. It is also important for clinicians to be aware that the vast majority of patients presenting with chest pain and minimal CAD do have an underlying abnormality of coronary vasomotion even if they do not have manifest ischemia on noninvasive testing. This is still not widely recognized amongst the cardiology community. Interventional cardiologists doing diagnostic procedures are critical in conveying this message to patients and referring physicians.

In addition, in a substantial proportion of patients with acute coronary syndromes, normal or near-normal coronary angiograms are found [18]. This condition is known as myocardial infarction with no obstructive coronary arteries (MINOCAs). Moreover, microvascular dysfunction is a major player in the no-reflow phenomenon in primary percutaneous coronary intervention (PCI) for STEMI [19]. However, in this issue, we focus on the microvascular dysfunction described above.

## 3. Etiology

Risk factors for MVD are the same as for CCS. Endothelial dysfunction is the first step in this process, and inflammation is central in the progression of the disease. Patients with coronary endothelial dysfunction are recognized to have significant health service use and morbidity as well as an increased risk of developing flow-limiting coronary artery disease and myocardial events, including death [20].

Additionally, recent studies have shown that especially vasospastic angina is associated with an early inflammatory coronary artery condition documented with the presence of low-grade inflammation-related endothelial dysfunction with resulting diffuse intimal thickening and impaired nitric oxide production [21]. Endothelial dysfunction, the precursor for CAD, is associated with MVD [22]. However, also nonendothelial-dependent vascular dysfunction is associated with the typical risk factors for atherosclerosis like aging [23], hypertension [24], diabetes [25], dyslipidemia, and insulin resistance [26]. The mechanisms underlying the development of MVD are thus multifactorial and only partly explained by current research.

In a small mechanistic study following PCI, both large- and small-vessel vasoconstriction were seen as manifested by a reduction in coronary conduit vessel diameter and in CBF. These effects were reversed by NTG. Serum levels of LDL were modestly related to the reduction of CBF and to the degree of NTG-induced vasodilatation of the coronary microvasculature [27].

## 4. Classification

In 2007, Camici and Crea presented a clinical classification with 4 subtypes of coronary microvascular dysfunction on the basis of the clinical settings in which it occurs: dysfunction occurring in the absence of CAD and myocardial diseases, dysfunction in the presence of myocardial diseases, dysfunction in the presence of obstructive epicardial CAD, and iatrogenic dysfunction [28].

The paper by Zelis et al. in the current issue sheds light on the coronary microvascular dysfunction in the presence of myocardial disease, i.e., aortic stenosis (AS) [29]. They describe the disadvantages of secondary cardiomyopathy in AS: diastolic dysfunction, insufficient capillary density, and diffuse fibrosis.

They refer to the area under the aortic (or, in situations of aortic stenosis, LV) curve during systole (systolic pressure time integral (SPTI)), which has been shown in animal models to have a very high and direct correlation with myocardial oxygen demand, even superior to the rate pressure product [30]. Furthermore, they refer to the diastolic pressure time integral (DPTI) which is an analog for “coronary perfusion pressure.” The ratio of these DPTI/SPTI balances supply and demand into a single unitless ratio, although this formulation ignores other factors such as arterial oxygen content and relative LV mass and wall tension [31].

After reviewing the available literature, they found that existing data support an increase in hyperemic flow after TAVI due to a change in the myocardial load line. This change is due to a reduction in wedge pressure, largely “reflecting” LV filling pressures that fall after AS has been treated.

## 5. Diagnosis and Methods

Established diagnostic tools for assessing microvascular disease are not readily available in most cath. labs, leaving many patients with no or a wrong diagnosis. Therefore, a growing part of the interventional cardiology community is looking for an available means to diagnose and quantify microvascular dysfunction to find the appropriate and accurate diagnosis for the individual patient.

For the last 2 decades, studies employing positron emission tomography (PET) have been used to describe the normal range of absolute myocardial blood flow (MBF, mL/min/g) and of coronary flow reserve (CFR). This is a measure of coronary circulatory capacity defined as the ratio of MBF during maximal coronary vasodilatation to baseline MBF [32].

The invasive methods presently used to assess microvascular function, CFR and Index of Microvascular Resistance (IMR), are operator dependent and are based on adenosine to induce hyperemia. In the current issue, Keullards et al. reviewed the new thermodilution-based method for the measurement of absolute coronary blood flow and microvascular resistance [33]. The measurements are easy to perform using the Rayflow® infusion catheter and Coroventis® software. The method is accurate, reproducible, and completely operator independent and has been validated noninvasively against the current golden standard for flow assessment: PET-CT [34].

It has recently been shown that a comprehensive invasive assessment of these patients at the time of coronary angiography can be performed safely and provides important diagnostic information that may affect treatment and outcomes [35]. This should be integrated in modern invasive diagnostics in that conventional stress testing is insufficient for identifying occult coronary abnormalities that are frequently present in patients with angina in the absence of obstructive CAD. A normal noninvasive test for ischemia does not rule out a nonobstructive coronary etiology of angina, nor does it negate the need for comprehensive invasive testing [36].

In addition to PET, intracoronary Doppler measurements are considered close to the gold standard for determining CFR. However, both types of examinations are associated with a certain load of ionizing radiation in addition to the obvious invasive nature of intracoronary Doppler measurements. In the search for less-invasive methods, transthoracic Doppler echocardiography (TTDE) has emerged as a robust method to assess CRF [37].

In the current issue of the journal, Bechsgaard and Prescott described the method of TTDE for assessing coronary flow velocity reserve [38] as an established method of assessment of coronary microvascular function with a well-documented prognostic significance [39]. They review the use of adenosine infusion as a microvascular dilatator by activation of A2A receptors yielding a 3- to 4-fold increase in coronary blood flow in a normal epicardial vessel [40]. Furthermore, they describe the ratio of hyperemic to resting coronary flow velocity, coronary flow velocity reserve (CFVR), as an established physiological estimate of coronary microvascular function, which is closely correlated with CFVR measured using an intracoronary Doppler guidewire in patients undergoing angiography for suspected obstructive CAD [41]. Dobutamine cMRI stress can yield useful information about wall motion abnormalities, and ischemia can be assessed using adenosine in INOCA subjects [42]. Due to the lack of radiation of both TTDE and cMRI, these methods might be particularly advantageous for young women with chest pain syndrome requiring diagnostic work-up.

## 6. Prognosis

Patients with MVD show persistence and even worsening of symptoms over time [43], and they constitute a therapeutic problem with considerable residual morbidity associated with functional limitations and reduced quality of life in addition to the increasing economic burden of the health authority system [44]. Impaired CFR is associated with increased mortality in patients with INOCA [45, 46]. Furthermore, impaired CFR without any concomitant impairment of regional or global left ventricular function has additional prognostic significance [47]. In a large study evaluating the prognostic significance of both stress myocardial blood flow (MBF) and myocardial flow reserve (MFR) and the ratio of stress to rest MBF [48], the researchers found MFR to be substantially more consistent, regardless of the choice of input function derivation method and the extraction model used [49].

The link between MVD and flow-mediated vasodilation is further underlined in regard to prognosis in a study evaluating hyperemic velocity, the stimulus for flow-mediated dilation. Hyperemic velocity was a significant risk marker for adverse cardiovascular outcomes. The prognostic value is additive to traditional risk factors and carotid intima-media thickness [50]. This suggests that microvascular dysfunction may be systemic, and that peripheral testing may be useful in diagnosis and prognosis.

The size of the problem and the lack of therapeutic intervention justify the increasing efforts to develop diagnostic tools and to identify new treatment strategies when assessing patients with INOCA.

## 7. Treatment

Reduced physical activity is one of the major avoidance behaviours in patients with coronary heart disease [51]. On the other hand, several studies have documented the positive effect of exercise training (ET) in this population [52]. Psychological morbidity with great impact on daily living is well known in both patients with cardiovascular disease and in patients with chest pain with no obvious physical disease. This includes patients with INOCA. These patients constitute a relatively large proportion of patients taken care of by the health authority system, indicating that this issue has economic consequences for the society that is not neglectable [2].

Therefore, a major end point in the treatment of these patients is symptom control [53].

In patients with MVA, lifestyle modifications such as smoking cessation and weight loss, which are known to improve endothelial dysfunction, are as essential as in the prevention and treatment of CAD [54]. Notably, exercise training has been shown to improve symptoms in this population [55]. A small observational study also indicates that an improvement in VO_2 peak_ is associated with increased CFR and improved endothelial function. Importantly [56], these effects were followed by an improvement in quality of life [57].

Long‐term treatment with carvedilol can significantly increase coronary flow reserve and reduce the occurrence of stress‐induced perfusion defects, suggesting a favorable effect of the drug on coronary microvascular function in patients with IDC [58]. Additionally, Neglia and coworkers showed a beneficial effect of perindopril on coronary blood flow after 6 months of perindopril treatment. This treatment has also been shown to improve myocardial blood flow and reverse remodeling in myocardial arterioles in spontaneous hypertensive rats [59]. A large multicentre, prospective, randomized, blinded outcome study evaluating intensive medical therapy including high-intensity statins, ACE-Is or ARBs, and aspirin, vs. usual care in 4422 symptomatic women with INOCA, (Women's IschemiA TRial to Reduce Events In Non-ObstRuctive CAD, (WARRIOR)), will probably give the answer if patients with MVD should be treated as patients with CAD [60].

The purpose of the CorMica trial was to evaluate whether an interventional diagnostic procedure (IDP) linked to stratified medicine improves health status in patients with INOCA. Patients without angiographical obstructive CAD (*n* = 151–39%) were immediately randomized 1 : 1 to the intervention group (stratified medical therapy) or the control group (standard care, IDP sham procedure). The IDP consisted of guidewire-based assessment of coronary flow reserve, index of microcirculatory resistance, and fractional flow reserve, followed by vasoreactivity testing with acetylcholine. The primary endpoint was the mean difference in angina severity at 6 months. The authors concluded that stratified medical therapy, including an IDP with linked medical therapy, was routinely feasible and improved angina in patients with no obstructive CAD [17]. This strategy leads to marked and sustained angina improvement and better quality of life at 1 year following invasive coronary angiography [61]. The findings from this trial underline the need for an extended diagnostic framework when evaluating patients with INOCA. The correct diagnosis is a prerequisite for proper medical therapy and lifestyle intervention to increase quality of life in this population.

## 8. Conclusions

Microvascular dysfunction is responsible for angina in a substantial number of patients admitted for coronary angiogram. Diagnostic options are very limited in most centers, although these patients may have significant effects from cardiovascular risk reduction programs and tailored medical treatment, both in terms of symptoms and prognosis. Interventional cardiologists must lead the expansion of testing for microvascular angina so that the patients and the referring clinician have the correct diagnosis, which will aid in improved quality of life in these subjects.

*Alf I. Larsen* *William F. Fearon* *Todd J. Anderson* *Nico Pijls*
